# Mortality Predictors in Patients with Severe COPD Exacerbation

**DOI:** 10.3390/jcm14093028

**Published:** 2025-04-27

**Authors:** Elena Cojocaru, Raluca Ecaterina Haliga, Gianina-Valentina Băcescu Ene, Cristian Cojocaru

**Affiliations:** 1 Morpho-Functional Sciences II Department, Faculty of Medicine, “Grigore T. Popa” University of Medicine and Pharmacy, 700115 Iași, Romania; elena.cojocaruu@umfiasi.ro; 2 Medical III Department, Faculty of Medicine, “Grigore T. Popa” University of Medicine and Pharmacy, 700115 Iași, Romania; cristian.cojocaru@umfiasi.ro; 3 Department of Pneumology, “Iuliu Hat, ieganu” University of Medicine and Pharmacy, 400332 Cluj-Napoca, Romania; bacescu.ene.giani.valentina@elearn.umfcluj.ro; 4 Pneumology Clinic, Clinical Hospital of Pneumophthisiology, 700115 Iași, Romania

**Keywords:** COPD, severe exacerbation, ICU, hematological indices, prognostic biomarkers

## Abstract

**Background:** Severe acute exacerbations of chronic obstructive pulmonary disease (AECOPD) are a leading cause of intensive care unit (ICU) admissions and in-hospital mortality. Several hematological inflammatory biomarkers, including neutrophil-to-lymphocyte ratio (NLR), platelet-to-lymphocyte ratio (PLR), monocyte-to-lymphocyte ratio (MLR), derived NLR (dNLR), and systemic immune-inflammation index (SII), have been proposed as markers of disease severity and mortality. **Methods:** A retrospective study was conducted on 104 ICU patients with AECOPD over a two-year period. We collected and analyzed clinical, demographic, and laboratory data. The hematological indices of the two groups—survivors (n = 39) and non-survivors (n = 65)—were compared to assess differences. We used t-tests, ANOVA, chi-square tests, and Mann–Whitney U tests to compare the groups. The factors that independently predicted mortality were identified using multivariate logistic regression. We examined survival differences using Kaplan–Meier analysis, and ROC curves were utilized to evaluate the predictive power of each biomarker. **Results:** Mortality was substantially predicted by higher SII (OR: 1.92, 95% CI: 1.24–3.08, *p* = 0.002) and NLR (OR: 2.89, 95% CI: 1.72–4.82, *p* < 0.001). Patients with NLR > 8.0 and SII > 1800 had significantly lower survival rates (log-rank *p* < 0.001), according to Kaplan–Meier analysis. SII (AUC = 0.79) and NLR (AUC = 0.82) were the best predictors of death, according to ROC analysis. **Conclusions:** In ICU-admitted AECOPD patients, NLR, MLR, PLR, dNLR, and SII are independent predictors of mortality. Due to their ease of evaluation and predictive capabilities, they should be included in ICU risk models for early interventions.

## 1. Introduction

Chronic obstructive pulmonary disease (COPD) exacerbations, especially severe acute ones that require intensive care unit (ICU) admission, have a high morbidity and mortality rate. The incidence varies across the world due to variations in healthcare systems and patient characteristics. COPD is the fourth leading cause of death globally, accounting for 3.5 million deaths in 2021 (5% of global mortality), despite earlier estimates that this ranking would not be reached until 2030 [1,2]. COPD is also the eighth leading cause of health deterioration (in disability-adjusted life years), and nearly 90% of mortality in individuals younger than 70 years is found in low- and middle-income countries. The rate of ICU admission in hospitalized patients with acute exacerbation of chronic obstructive pulmonary disease (AECOPD) varies across studies. ICU admission varies from 2% to 19%, with an in-hospital mortality of 20–40% [3]. Other studies have found ICU admission rates to be 32.25% [4], 33.1% [5], and 51.6% [6], illustrating the severity of the condition. That there is heterogeneity in patient outcomes emphasizes the need for improved risk stratification so that high-risk individuals are identified early and optimized management approaches are ensured. Systemic inflammation is a feature of COPD exacerbation and results in airway obstruction, lung parenchymal injury, and multi-organ involvement.

Systemic inflammation is a hallmark of COPD exacerbation and causes airway obstruction, lung parenchymal injury, and multi-organ disease. ICU scoring systems such as APACHE II (Acute Physiology and Chronic Health Evaluation II) and SOFA (The Sequential Organ Failure Assessment score) provide an overall estimate of the severity of critical illness but do not measure individually the contribution of systemic inflammation to disease outcome. Recent evidence suggests that inflammatory hematological indices like neutrophil-to-lymphocyte ratio (NLR), platelet-to-lymphocyte ratio (PLR), monocyte-to-lymphocyte ratio (MLR), derived neutrophil-to-lymphocyte ratio (dNLR), and systemic immune-inflammation index (SII) may serve as objective biomarkers predicting mortality [7,8]. As systemic inflammation is a key component of the pathophysiology of COPD, studying these indices may provide important insights into disease progression to inform risk stratification and patient-tailored management strategies. The indices, which can be measured from standard blood work, are a cost-effective and readily accessible method of ascertaining inflammatory burden and guiding clinical decision-making. These biomarkers are of clinical interest for disease progression assessment and the risk of exacerbation in COPD subjects, besides their role in predicting mortality. Derived from routine blood tests, they reflect levels of inflammation and oxygenation status, both essential for disease management.

Despite growing interest, the prognostic value of these hematological markers in AECOPD patients requiring ICU admission remains under-studied. The aim of this study is to determine whether these biomarkers are independently adequate for predicting mortality in ICU-admitted AECOPD patients and thereby capable of providing valuable information for early risk stratification and management planning.

## 2. Materials and Methods

### 2.1. Study Design and Population

This is a retrospective observational cohort study of patients with AECOPD who required intensive care at the Clinic of Pulmonary Diseases, Iași, Romania, from 1 January 2023 to 31 December 2024.

**Figure 1:**
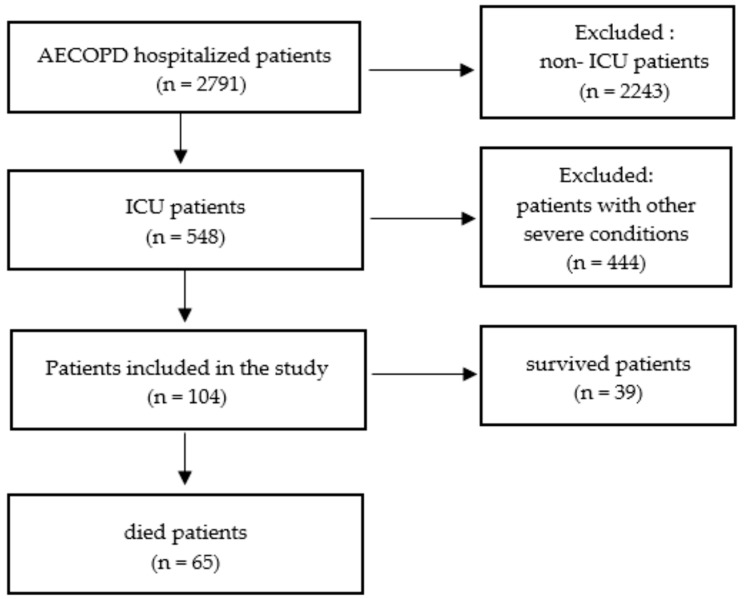
PRISMA flowchart of patient selection for study; AECOPD—acute exacerbations of chronic obstructive pulmonary disease; ICU—intensive care unit; n—number of patients.

The study period was chosen intentionally to exclude potential confounding effects of hospitalizations as a result of Severe Acute Respiratory Syndrome Coronavirus 2 infections prior to 1 January 2023. The coronavirus disease 2019 (COVID-19) pandemic significantly altered hospitalization practices and the clinical approach to respiratory illness, and inclusion of data during this time may lead to confounding biases. Thus, the investigation was based on post-pandemic admissions to keep the results representative for non-COVID-19-related AECOPD mortality risk, excluding biases due to pandemic-related inequalities in treatment. The investigation was performed in accordance with the ethical guidelines in the Declaration of Helsinki and was approved by the Ethics Committee of the Clinical Hospital of Pneumophthisiology in Iași, Romania (Approval No. 120/06.03.2025). The included patients were AECOPD patients over 18 years old with a diagnosed COPD, according to The Global Initiative for Chronic Obstructive Lung Disease (GOLD) criteria, who required ICU for treatment. In total, 2791 patients were admitted with AECOPD within the study time. In the unit where the study was conducted, there is no dedicated respiratory ICU, and therefore, patients experiencing severe exacerbations of COPD were directly transferred to the general ICU. Among these, 548 required ICU admission and the remaining 2243 were managed in other wards of the hospital. Out of the 548 patients in the ICU, 444 were excluded since they had other dominant comorbidities (e.g., malignancies, end-stage organ failure, autoimmune disorders), and 104 patients with AECOPD stayed in the ICU. Of these, 65 patients died, while 39 survived and were discharged (Figure 1).

### 2.2. Data Collection

Demographic, clinical data and hematologic analyte values were extracted from electronic medical records. Variables collected included age, sex, comorbidities such as hypertension, diabetes and cardiovascular disease, length of ICU stay, mechanical ventilation requirements and treatment provided.

### 2.3. Blood Test Measurement

Venous blood samples were collected upon ICU admission and analyzed within two hours by trained phlebotomists. All samples were drawn following a standardized protocol after a fasting period of nine hours. The hematological analytes were measured on the Sysmex XN-550 hematology analyzer. Platelets were counted using electrical impedance (Coulter principle), and white blood cells were differentiated on the basis of fluorescence intensity (proportional to the concentration of nucleic acids), forward scatter (reflecting the size of the cells), and side scatter (a function of the cell granularity and complexity). The hemoglobin concentrations were measured colorimetrically using the hemoglobin sodium lauryl sulfate method, a cyanide-free technique. C- reactive protein (CRP) levels were measured as a marker of systemic inflammation using the Cobas Integra 400 Plus analyzer by turbidimetric immunoassay.

The hematological indices were calculated using standard formulas. NLR was determined by dividing the neutrophil count by the lymphocyte count, PLR was calculated as the platelet count divided by the lymphocyte count, MLR was determined by dividing monocytes by lymphocytes, SII was computed as the platelet count multiplied by the neutrophil count and divided by the lymphocyte count, and dNLR was calculated as the neutrophil count divided by the white blood cell count minus neutrophils.

### 2.4. Statistical Analyses

Statistical analysis was performed with MedCalc Statistical Software, version 23.1.7. *T*-tests and Mann–Whitney U tests were employed for continuous variables, while chi-square tests were employed for categorical variables. ANOVA was employed for comparing more than two groups. Logistic regression models were employed to ascertain independent predictors of mortality after adjusting for confounders such as age, comorbidities, and ICU length of stay. Survival differences were compared using Kaplan–Meier survival analysis, and ROC curves were generated to calculate the discriminatory capability of every biomarker. Any *p*-value below 0.05 was statistically significant.

## 3. Results

There were 104 ICU-admitted patients with severe AECOPD in the study. Of those, 65 (62.5%) died in hospital, and 39 (37.5%) survived and were discharged. The somatic and disease characteristics, along with the main composite markers, are summarized in Table 1. To identify factors associated with mortality risk, the collected data were compared between these two groups. The findings provide insights into potential predictive factors associated with the risk of death.

The mean age of the non-survivors was significantly higher (74.9 ± 8.9 years) than that of the survivors (69.2 ± 7.2 years, *p* = 0.001), suggesting that age is an independent risk factor for mortality in AECOPD. Sex distribution between the two groups was not significantly different (*p* = 0.16), suggesting gender was not an independent predictor of mortality. In the deceased group, 84.6% (55 patients) were smokers or ex-smokers with a history of more than 20 pack-years, while the remaining 15.4% either did not smoke or had no recorded smoking history (10 patients).

Hospitalization was less in non-survivors (10.9 ± 6.9 days) compared with survivors (14.5 ± 4.6 days, *p* < 0.05). This suggests that mortality in AECOPD can be associated with early and severe worsening but not prolonged ICU durations leading to fatal complications. Cardiovascular comorbidities were highly prevalent with coronary heart disease, heart failure, or hypertension being present in 84.6% of the non-survivors and 79.5% of survivors but again statistically not differentiated (*p* = 0.69). The remaining conditions, diabetes mellitus and chronic kidney disease, were somewhat equally distributed by group. These results indicate that cardiovascular disease is a common comorbidity in COPD patients admitted to the ICU. The distribution of urban and rural residency did not differ significantly between groups (*p* = 0.6). The distribution of COPD subgroups did not show significant differences between non-survivors and survivors (*p*-values ranging from 0.49 to 0.65). Group E was the most prevalent classification in both groups (64.6% in non-survivors vs. 56.4% in survivors). No significant differences in treatment regimens were found between survivors and non-survivors (*p* = 0.39–0.95), suggesting that factors like disease severity and systemic inflammation were more influential in survival.

In general, there were no statistically significant differences between the two groups regarding demographic characteristics, except for the higher age of non-survivors and the longer hospital stay in survivors. These findings led us to investigate possible haematological alterations that may be related to mortality risk and cardiovascular comorbidities.

**Figure 2:**
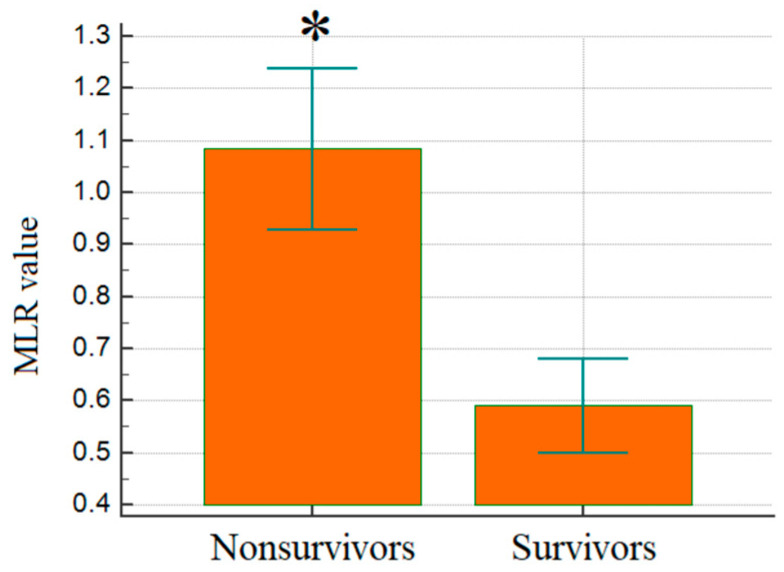
Comparison of mean MLR levels between groups; *p* < 0.001. MLR: monocyte-to-lymphocyte ratio; *: statistical significance.

The mean platelet volume-to-platelet count ratio (MPV/PC) was significantly different (0.06 ± 0.04 in non-survivors vs. 0.06 ± 0.02 in survivors, *p* < 0.001), though the clinical implications require further exploration. PLR was elevated in non-survivors (300.4 ± 214.4) vs. survivors (143.5 ± 72.0) (*p* < 0.001), reinforcing the potential link between platelet activation and poor prognosis. Although both groups had similar platelet-to-hemoglobin ratio (PHR) values, the statistical significance (*p* = 0.001) suggests potential clinical relevance. Comparative analysis of data revealed an extreme difference in MLR with considerably higher values in non-survivors compared to survivors (1.1 vs. 0.6, *p* < 0.001), suggesting monocyte-driven inflammation as a contributor to increased mortality (Figure 2).

**Figure 3:**
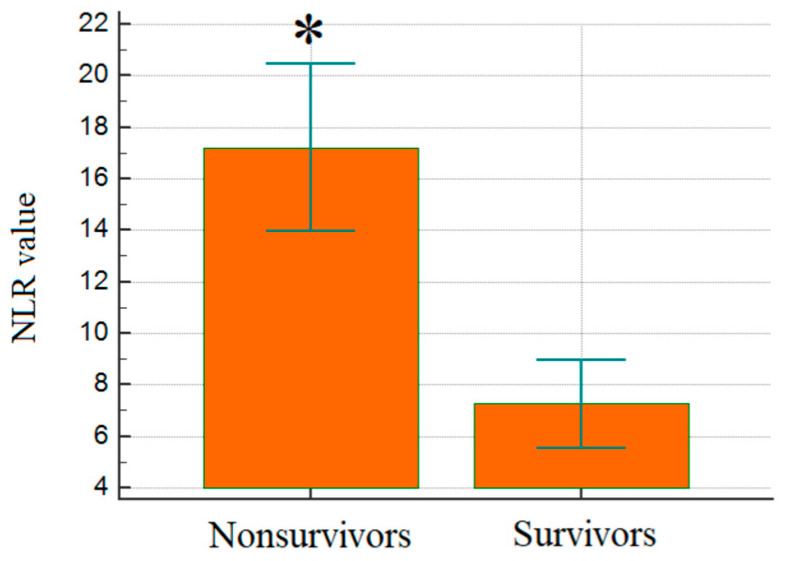
Comparison of mean NLR levels between groups; *p* < 0.001. NLR: neutrophil-to-lymphocyte ratio; *: statistical significance.

The NLR was similarly a powerful predictor of mortality among the studied cohort (Figure 3). The NLR levels were considerably elevated in non-survivors compared to survivors (23.0 vs. 8.7, *p* < 0.001). This underlines the systemic inflammation’s involvement in the severity of disease and risk of mortality. Consistent with existing literature that has validated NLR as an independent mortality prognostic factor [9], our findings also affirm its utility as a predictor in critically ill COPD patients requiring intensive care. Most notably, in survivors, the values of NLR recorded here were higher than those previously documented in the general population.

**Figure 4:**
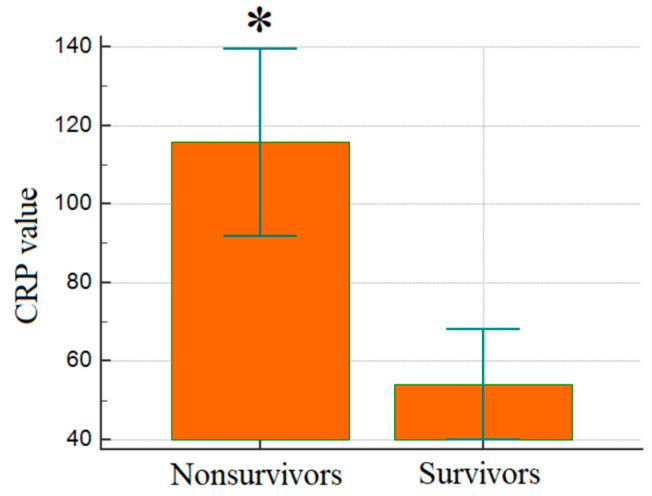
Comparison of mean CRP levels between groups; *p* < 0.001. CRP: C-reactive protein; *: statistical significance.

Figure 4 shows that CRP levels were significantly higher in non-survivors (115.7 mg/L ± 95.8) compared to survivors (54.0 mg/L ± 42.5) (*p* < 0.001), emphasizing the link between inflammation and adverse outcomes in ICU patients with COPD.

**Figure 5:**
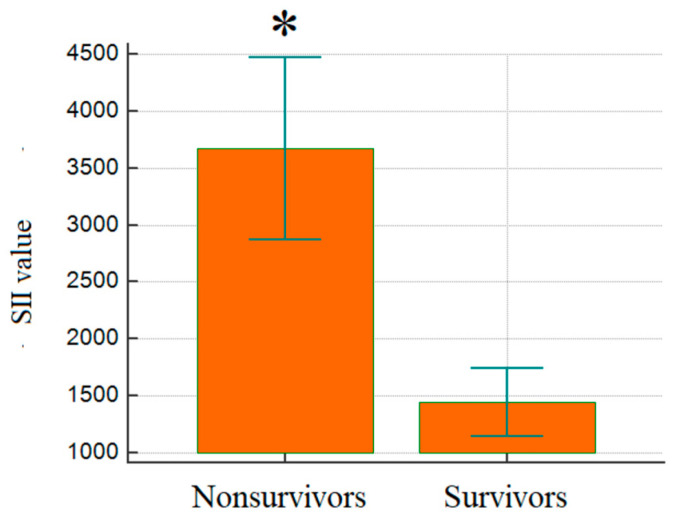
Comparison of mean SII levels between groups; *p* < 0.001. SII: systemic immune-inflammation index; *: statistical significance.

Figure 5 shows the difference in SII in groups, and the values are considerably higher in the deceased group. The SII was considerably elevated in non-survivors (3671.3 ± 3220.9) compared to survivors (1444.2 ± 933.3) (*p* < 0.001), which emphasizes its prognostic value in critical illness.

Similar statistical variations were observed between the two groups for the PLR, MPV/PC, and PHR. The PLR was higher among the deceased than in the documented cut-off of 180 and 226.67 found in other research studies [10,11].

A correlation matrix was used to determine variables that are correlated with risk of mortality. We focused on analyzing the composite indices outlined in Table 1, and age and plasma CRP levels. The results indicated that the factors that were positively correlated with mortality were age (correlation coefficient, r = 0.34), CRP (r = 0.35), NLR (r = 0.4), MLR (r = 0.41), and SII (r = 0.38). Specifically, NLR and MLR show a moderately strong positive correlation with mortality, suggesting that higher levels of these markers are associated with an increased risk of death. To determine the impact of variables on mortality, a logistic regression model was created, in which all correlations were statistically significant (*p* < 0.05).

**Figure 6:**
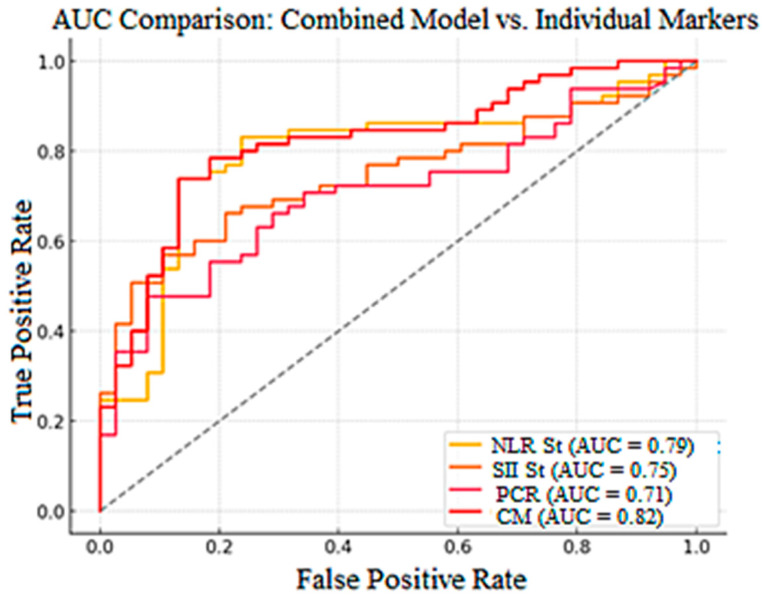
Receiver operating characteristic (ROC) curve comparing the predictive performance of the combined model and individual biomarkers. AUC—area under the curve; NLR St—neutrophil-to-lymphocyte ratio standardized; SII St—systemic immune-inflammation index standardized; CRP—C-reactive protein; CM—combined model.

A receiver operating characteristic (ROC) curve was drawn to verify the efficiency of the model, indicating that the model was effective (AUC = 0.79, 0.75, 0.71, 0.82) in discriminating between surviving and deceased patients. The ROC curve indicates that the chosen variables in the model are significant in predicting mortality (Figure 6).

These above analyses ultimately led to the establishment of risk categories based on the likelihood of mortality, as outlined in Table 2. Clinical decision-making may be informed by risk stratification based on objective data, and risk stratification may possibly guide future studies regarding the impact of various therapeutic interventions in managing patients with severe exacerbations of COPD.

**Figure 7:**
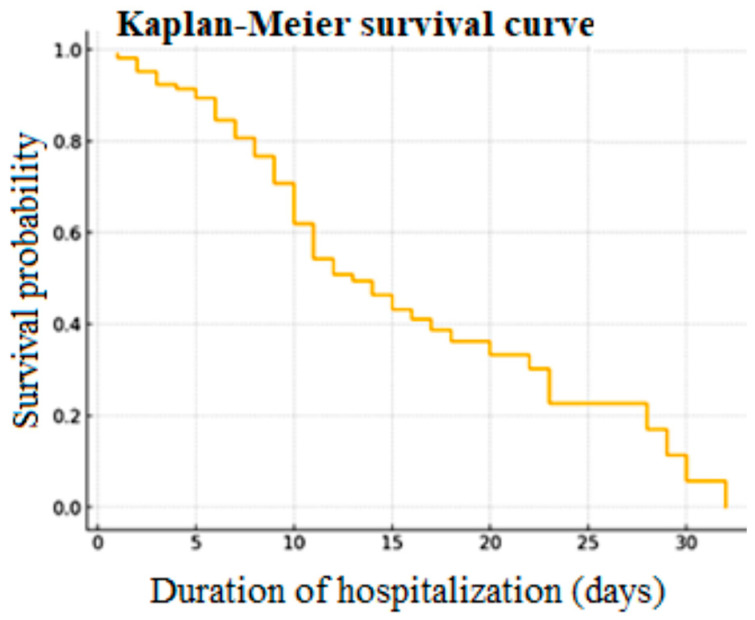
Kaplan–Meier survival curve.

Kaplan–Meier survival curve analysis by days of hospitalization (Figure 7) shows that the critically ill are at great risk of death early in the days of admission and that 50% of the deaths happen in the first 10 days. Those patients who survive this phase of high risk have a greater chance of survival; however, the prolonged hospitalization also has an excess mortality risk. Severe prognosis is strongly associated with cardiovascular comorbidities.

We conducted an ANCOVA (Analysis of Covariance) to compare the means of the groups (e.g., mortality status: survivors and non-survivors) for the variables of study, adjusting for age and gender. We analyzed whether the means of the variables differed significantly between the groups after adjustment for the covariates. The results showed that PCR (F = 15.59, *p* < 0.001), M/L (F = 13.56, *p* < 0.001), N/L (F = 13.26, *p* < 0.001), and SII (F = 12.25, *p* < 0.001) were all significant predictors of mortality. Gender and age did not significantly affect the biomarkers in this research.

## 4. Discussions

NLR, MLR, PLR, dNLR, and SII have also been found to be good biomarkers in diagnosing AECOPD. Being easily available and cost-effective parameters, they are extremely significant in detecting patients at high risk of in-hospital adverse outcomes. Research has indicated that elevated levels of these indices are associated with increased inflammation and poorer clinical outcomes, including increased risk of ICU admission and mortality. Their prognostic utility in AECOPD is further supported by their association with other inflammatory markers and their capacity to reflect systemic inflammation during exacerbations.

Our results support existing research that has pointed out the prognostic significance of NLR in COPD exacerbations, especially in the ICU [12,13,14]. It is a steady and reliable marker and may be applied on a regular basis to predict outcomes and ascertain inflammation in AECOPD patients. NLR is associated with systemic inflammation, a distinguishing feature of COPD exacerbation, and may be employed to assess disease severity and response to treatment [15,16,17].

The identification of mortality predictors in patients with exacerbations of COPD remains a current challenge, given the increasing mortality associated with this condition. Various factors have been investigated to date, and it appears clear that the risk of mortality is elevated not only during hospitalization but also immediately after discharge. Pulmonary function, exacerbation frequency, and age are variables that have been extensively studied [18]. On the other hand, due to comorbidities, particularly cardiovascular diseases, determining the exact cause of death is often challenging. In general, in-hospital mortality among patients with COPD exacerbations is estimated to range from 2.5% to 14% [19], while mortality in ICUs can be as high as 30% [20].

Recently, several composite variables have been studied in relation to the risk of unfavorable outcomes in respiratory infections, such as COVID-19. 

MLR has been explored as a novel hematologic marker of systemic inflammation [21]. The mechanism by which monocytes may suppress lymphocyte activation is supported by the presence of systemic inflammation and has been investigated in connection with certain cardiovascular diseases. However, results to date remain inconclusive. In our study, MLR is strongly associated with mortality in patients with COPD exacerbations, suggesting that it could serve as a robust independent predictor of mortality in this patient population.

NLR is an established prognostic factor for mortality in various diseases, although its optimal cut-off value remains debated. NLR has been proposed as a surrogate index of systemic inflammation. Elevated NLR indicates an exaggerated neutrophilic response, which is typically related to tissue damage and poor outcomes in COPD exacerbations. NLR is an early sign of pathological conditions or processes including cancer, atherosclerosis, infection, inflammation, psychiatric disorder, and stress. In this study, both the presence of infections and background cardiovascular pathology result in the impact on NLR. Studies have shown that NLR values > 4.5 are a standalone predictor of cardiovascular diseases [22], and the same has been observed in this study as well. Looking at the results obtained and previous studies, we can hypothesize that increased NLR values among deceased patients are most probably due to various factors like infection, stress, metabolic derangements, exogenous steroids, and cardiovascular comorbidities.

In one study [23], NLR was found to be markedly elevated in COPD and bronchopulmonary neoplasms compared to non-COPD patients. Levels above 18 are considered critical [24], and were noted in the group of deceased patients, whereas in the survivor group, the levels were in line with decreased stress. A retrospective analysis assessing the correlation of NLR with in-hospital mortality in patients presenting with exacerbation of COPD identified that a cut-off of 6.9 was related to a sensitivity of 60.87% and specificity of 73.29% in identifying mortality risk in patients under observation [25]. The findings of this study suggest that an increase in NLR, as a marker of systemic inflammation, is directly proportional to mortality, and thus this composite marker can prove to be an effective tool for the identification of high-risk death patients.

Low PLR values are suggestive of the absence of a pro-inflammatory and thrombotic status. Although elevated PLR values in both groups indicate the presence of inflammation and a prothrombotic state, significantly higher values in the deceased patient group suggest that this marker may be valuable in identifying the prognosis of patients with cardiovascular risk and severe COPD exacerbation. In one study, a PLR ≥ 235 was significantly associated with increased 90-day mortality in patients experiencing AECOPD. The study reported that this PLR threshold had a sensitivity of 63% and specificity of 74% for predicting 90-day mortality [26]. These findings suggest that elevated PLR can serve as a prognostic marker in AECOPD management.

It is well known that the risk of death in patients with severe COPD exacerbation requiring intensive care support is high. The current analysis revealed that, although the median survival time is 10 days, most patients survive the first 7 days (83.6%). We found that the most likely period of death for these patients occurs between days 7 and 14. Advanced age and serum CRP levels, consistent with other studies, are the strongest independent predictive factors of mortality. Although SII demonstrated a significant correlation with mortality, it does not appear to be a superior predictive marker in this study, and it remains relatively unexplored in the published literature to date.

Strong clinical evidence supports that systemic inflammatory response is a key determinant of mortality risk for critically AECOPD patients who are admitted to the ICU. Hematologic markers provide useful information on the immune dysregulation, inflammatory burden, and coagulation abnormality that underlies AECOPD development in ICUs. Of these markers, SII is a composite biomarker that captures the hyperinflammatory state, thrombogenicity, and immune suppression of critically AECOPD patients. Elevated SII is associated with neutrophilia, which amplifies proinflammatory cytokine secretion and oxidative stress-induced alveolar injury, leading to acute respiratory failure. Thrombocytosis, which occurs simultaneously, exacerbates microvascular thrombosis, endothelial dysfunction and impaired perfusion, leading to multiorgan failure. Lymphopenia is also a marker of dysregulated adaptive immunity, with patients being susceptible to secondary infections, prolonged ICU stay, and mortality. NLR, as a marker of systemic inflammation and immune exhaustion, is significantly elevated in patients with AECOPD, in which excessive neutrophilic infiltration and lymphopenia create a sustained proinflammatory status, augmenting tissue injury and respiratory failure. High NLR levels have been shown to predict increased in-hospital mortality with increasing levels of elevation associated with sepsis, mechanical ventilation, and multiorgan failure. Similarly, PLR as a marker of hypercoagulability and chronic inflammation was also implicated in venous thromboembolism risk, endothelial dysfunction, and cardiovascular events in AECOPD patients. Elevated PLR was also among the important predictors of in-hospital mortality, and its role in early prognostication was noted. The MLR, reflecting monocyte-driven inflammatory responses, provides additional prognostic value by quantifying excessive cytokine activation and immune suppression, which impair tissue repair mechanisms and antimicrobial defense. Elevated MLR is associated with higher in-hospital mortality, emphasizing its relevance in evaluating disease severity and immune dysfunction.

Study limitations comprise a small patient sample size, which can limit generalizability. As a retrospective cohort, this study is prone to biases, such as selection and information bias, affecting causal conclusions. Having been conducted at a single center in Romania, the findings may not be widely generalizable to other health care settings. Despite adjustment for major comorbidities, other unmeasured factors may influence outcomes. Additionally, the study is focused on short-term mortality, and long-term follow-up would be required to ascertain long-term implications.

All studied parameters may constitute a multiparametric and multidimensional biomarker profile in mortality risk assessment for AECOPD patients hospitalized in an ICU. SII, NLR, PLR, and MLR are interdependent yet unique pathophysiologic processes of hyperinflammation, immune suppression, thrombogenicity, and hypoxia-induced organ damage. Their integration into clinical risk models could enhance early mortality prediction, guide therapeutic interventions, and personalize resource allocation in critical care units. In the future, establishing uniform cut-off values and conducting prospective validation studies will be essential in order to consolidate their roles in precision medicine and individualized critical care management of AECOPD patients.

## 5. Conclusions

Our research underscores the predictive value of hematologic markers such as CRP, NLR, MLR, and SII in assessing the risk of mortality among ICU-admitted patients with severe COPD exacerbations. Our findings confirm the critical role of systemic inflammation in determining patient outcomes and suggest that these easily measurable biomarkers could be incorporated into clinical risk stratification tools. Compared to traditional severity scores, NLR, MLR and SII are cost-effective and reliable tools for the early identification of high-risk patients, allowing time for interventions. The present study provides good evidence for the clinical utility of these biomarkers and paves the way for further prospective work to validate their usefulness in the management of treatment strategies and the improvement of patient outcomes in exacerbations of severe COPD. That these indices can be included in a multiparametric model to foresee mortality in ICU patients is a significant advance in personalized medicine for COPD patients.

## Figures and Tables

**Table 1:** Baseline characteristics of all patients according to outcome.

Characteristics	Non-Survivors (n = 65)	Survivors (n = 39)	*p* Value
Age (years)	74.9 ± 8.9	69.2 ± 7.2	0.001
Male	54 (83.1%)	27 (69.2%)	0.16
Hospitalization (days)	10.9 ± 6.9	14.5 ± 4.6	<0.05
ICU duration (days)	6.8 ± 6.7	5.6 ± 2.5	0.29
COPD groups Group A Group B Group E	2 (3.1%) 21 (32.3%)	1 (2.6%) 16 (41.0%)	0.65 0.49
	42 (64.6%)	22 (56.4%)	0.53
Cardiovascular comorbidities	55 (84.6%)	31 (79.5%)	0.69
Cardiac failure	32 (49.2%)	12 (30.8%)	0.1
Hypertension	29 (44.6%)	10 (25.6%)	0.08
Diabetes	11 (16.9%)	10 (25.6%)	0.41
Therapy LAMAs LABAs/LAMAs ICS/LABAs ICS/LABAs/LAMAs	8 (12.3%)	2 (5.1%)	0.39
	15 (23.1%) 21 (32.3%) 21 (32.3%)	12 (30.8%) 12 (30.8%) 13 (33.3%)	0.52 0.95 0.91
NLR	17.2 ± 13.1	7.3 ± 5.3	<0.001
PLR	300.4 ± 214.4	143.5 ± 72.0	<0.001
MLR	1.1 ± 0.6	0.6 ± 0.3	<0.001
MPV/PC ratio	0.06 ± 0.04	0.06 ± 0.02	<0.001
PHR	17.9 ± 9.5	17.6 ± 5.7	0.001
SII	3671.3 ± 3220.9	1444.2 ± 933.3	<0.001
CRP (mg/L)	115.7 ± 95.8	54.0 ± 42.5	<0.001
Lactate (mmol/L)	2.26 ± 1.53	0.5 ± 0.29	<0.001
pH	7.25 ± 0.13	7.35 ± 0.12	<0.001
Lactate to dNLR ratio	0.48 ± 0.44	0.19 ± 0.18	<0.001

n—number of patients; ICU—intensive care unit; COPD—chronic obstructive pulmonary disease; LAMAs—long-acting muscarinic antagonists; LABAs—long-acting beta 2-agonists; ICS—inhaled corticosteroids; NLR—neutrophil-to-lymphocyte ratio; PLR—platelet-to-lymphocyte ratio, MLR—monocyte-to-lymphocyte ratio; MPV/PC ratio—mean platelet volume-to-platelet count ratio; SII—systemic immune-inflammation index; PHR—platelet-to-hemoglobin ratio; CRP—C-reactive protein.

**Table 2:** Risk stratification and mortality rates in patients with severe COPD exacerbations.

Risk Category	Mortality Rate
Low mortality risk (<30%)	16.7%
Moderate mortality risk (30–70%) High mortality risk (>70%)	41.7% 95.9%

## Data Availability

The database used and analyzed during the current study is available from the corresponding author on reasonable request.
